# Mechanical guidance of self-condensation patterns of differentiating progeny

**DOI:** 10.1016/j.isci.2022.105109

**Published:** 2022-09-27

**Authors:** Takahisa Matsuzaki, Yuko Shimokawa, Hiroyuki Koike, Masaki Kimura, Yuma Kawano, Nao Okuma, Ryuzo Kawamura, Yosuke Yoneyama, Yasuro Furuichi, Fumihiko Hakuno, Shin-Ichiro Takahashi, Seiichiro Nakabayashi, Satoshi Okamoto, Hiromitsu Nakauchi, Hideki Taniguchi, Takanori Takebe, Hiroshi Y. Yoshikawa

**Affiliations:** 1 Center for Future Innovation, Graduate School of Engineering, Osaka University, 2-1, Yamadaoka, Suita, Osaka 565-0871, Japan; 2 Department of Applied Physics, Graduate School of Engineering, Osaka University, 2-1, Yamadaoka, Suita, Osaka 565-0871, Japan; 3 Division of Strategic Research and Development, Graduate School of Science and Engineering, Saitama University, Shimo-Okubo 255, Sakura-Ku, Saitama 338-8570, Japan; 4 Department of Chemistry, Saitama University, Shimo-okubo 255, Sakura-ku, Saitama 338-8570, Japan; 5 Department of Pediatrics, University of Cincinnati College of Medicine, 3333 Burnet Avenue, Cincinnati, OH 45229- 3039, USA; 6 Division of Gastroenterology, Hepatology and Nutrition, Developmental Biology, Center for Stem Cell and Organoid Medicine (CuSTOM), Cincinnati Children’s Hospital Medical Center, 3333 Burnet Avenue, Cincinnati, OH 45229-3039, USA; 7 Institute of Research, Division of Advanced Multidisciplinary Research, Tokyo Medical and Dental University, 1-5-45 Yushima, Bunkyo-Ku, Tokyo 113-8510, Japan; 8 Departments of Animal Sciences and Applied Biological Chemistry, Graduate School of Agriculture and Life Sciences, the University of Tokyo, Bunkyo-Ku, Tokyo 113-8657, Japan; 9 Department of Health Promotion Sciences, Graduate School of Human Health Sciences, Tokyo Metropolitan University, 1-1 Minami-Osawa, Hachioji, Tokyo, Japan; 10 Department of Regenerative Medicine, Graduate School of Medicine, Yokohama City University, Kanazawa-Ku 3-9, Yokohama, Kanagawa, 236-0004, Japan; 11 Division of Regenerative Medicine, Center for Stem Cell Biology and Regenerative Medicine, The Institute of Medical Science, University of Tokyo, 4-6-1, Shirokanedai, Minato-Ku, Tokyo 108-8639, Japan; 12 Institute for Stem Cell Biology and Regenerative Medicine, School of Medicine, Stanford University, Stanford, CA 94305, USA; 13 Center for Stem Cell Biology and Regenerative Medicine, Institute of Medical Science, University of Tokyo, Minato-Ku, Tokyo, Japan

**Keywords:** Mechanobiology, Biomechanical engineering, Biomaterials

## Abstract

Spatially controlled self-organization represents a major challenge for organoid engineering. We have developed a mechanically patterned hydrogel for controlling self-condensation process to generate multi-cellular organoids. We first found that local stiffening with intrinsic mechanical gradient (*IG* > 0.008) induced single condensates of mesenchymal myoblasts, whereas the local softening led to stochastic aggregation. Besides, we revealed the cellular mechanism of two-step self-condensation: (1) cellular adhesion and migration at the mechanical boundary and (2) cell-cell contraction driven by intercellular actin-myosin networks. Finally, human pluripotent stem cell-derived hepatic progenitors with mesenchymal/endothelial cells (*i.e.,* liver bud organoids) experienced collective migration toward locally stiffened regions generating condensates of the concave to spherical shapes. The underlying mechanism can be explained by force competition of cell-cell and cell-hydrogel biomechanical interactions between stiff and soft regions. These insights will facilitate the rational design of culture substrates inducing symmetry breaking in self-condensation of differentiating progeny toward future organoid engineering.

## Highlights

• Hydrogels with mechanical patterns control self-condensation of cells
• *In vivo*-inspired local stiffening drives condensation of cells toward stiff region
• Mechanical gradients produce a two-step self-condensation process of cells
• Morphological control of liver buds organoid and the constituents by mechanical patterns

## Graphical abstract

**Figure:**
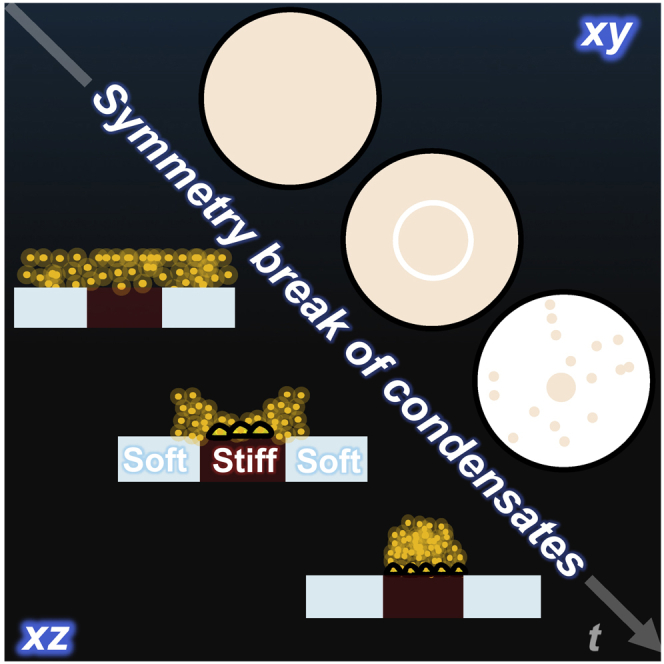

## Introduction

Bioengineering for shaping cell aggregates into tissue architecture has attracted much attention due to providing insights into the mechanism of organ development and the technical ease of clinical transplantation (Haraguchi et al., 2012; Lancaster and Knoblich, 2014; Matsusaki et al., 2013; Sasai, 2013; Yin et al., 2016). For example, as an artificially and mechanically assembled approach, complex three-dimensional tissues with morphological diversity have been achieved by various engineering techniques, such as automatic inkjet cell printing (Matsusaki et al., 2013) and stacked cell sheets (Haraguchi et al., 2012). Moreover, self-organization methods are promising means to obtain miniaturized organs (organoids) (Lancaster and Knoblich, 2014; Sasai, 2013; Yin et al., 2016). These methods utilize autonomous, collective cellular behaviors by mimicking organogenesis and regenerative processes *in vivo* (Lancaster and Knoblich, 2014; Sasai, 2013; Simunovic and Brivanlou, 2017; Yin et al., 2016). For example, we successfully established self-organizing endothelial networks that formed human-induced pluripotent cell (iPSC)-derived liver buds, which finally became vascularized and functional after transplantation (Takebe et al., 2013). Here, the formation of organ buds was initiated by a “self-condensation” step where 3D cell condensates are autonomously formed from mixtures of dissociated cells on a 2D hydrogel substrate. This morphogenetic transition from 2D to 3D led by mesenchymal cells can also be found in the early step of 3D liver bud development, where delamination from an endodermal sheet-like tissue occurs (Matsumoto et al., 2001). In addition, we previously revealed that mechano-responses of mesenchymal stem cells (MSCs) play a critical role in inducing self-condensation (Takebe et al., 2015). We found that MSC-driven self-condensation could be maximized by using a hydrogel substrate with a moderate mechanical property (Young’s modulus, *E*) (Takebe et al., 2015). It is known that MSCs exhibit various functions (*e.g*., contraction, adhesion, and differentiation) that are sensitive to the mechanics of their extracellular environments (Engler et al., 2006). These results indicate that fine-tuned extracellular mechanics is indispensable for organ bud development as well as other mechano-sensitive cell functions (Discher et al., 2005).

However, so far, most organoid studies used a homogeneous hydrogel substrate (Lancaster and Knoblich, 2014; Sasai, 2013), which is substantially different from heterogeneous mechanical fields *in vivo*. Based on further recent *in vivo* studies, locally stiffened areas in a soft environment play a crucial role in the collective cellular migration, which finally leads to complex morphogenesis of early (Moore et al., 1995) and later (Eiraku et al., 2011; Koser et al., 2016) embryos. Thus, studies of self-condensation on a locally stiffened substrate attract significant interest as a system inspired by a locally stiffened environment *in vivo* (*in vivo*-inspired systems). Furthermore, one can expect that mechanical patterns and gradients of hydrogel substrate may allow for the regulation of global morphology of cell condensates, which were limited to spheroids on a homogeneous hydrogel substrate. Forming asymmetric tissue morphologies is one of the critical steps of various organ development (*e.g.*, gastrulation). Thus, regulation of self-condensation steps by using a patterned hydrogel substrate can provide biophysical insights into the underlying mechanism of organogenesis toward further maturation of organoids.

In this report, we evaluated the impact of mechanical patterns of the underlying hydrogel substrate on self-condensation of mesenchymal mechano-responsible cell lines, including MSCs, which play a crucial role in an organoid generation according to Takebe’s protocol. Various patterned hydrogel substrates, such as an *in vivo*-inspired, locally stiffened substrate, were prepared by a photochemical reaction of hydrogel (Tse and Engler, 2010). We systematically investigated the self-condensation of mesenchymal cells on various mechanical patterns and identified the biomolecular mechanism. For lateral and axial morphological characterization of condensates, mechano-responsible, mammalian mesenchymal progeny expressing fluorescence cytoskeletal fiber was used. We also demonstrated the impact of the patterned substrates on the self-condensation of liver buds based on three different cell lines, MSCs, human umbilical vein endothelial cells (HUVECs), and induced pluripotent stem cell-derived hepatic endoderm (iPSC-HE).

## Results

### Design of mechanical patterning on hydrogels by photochemical reactions

**Figure 1:**
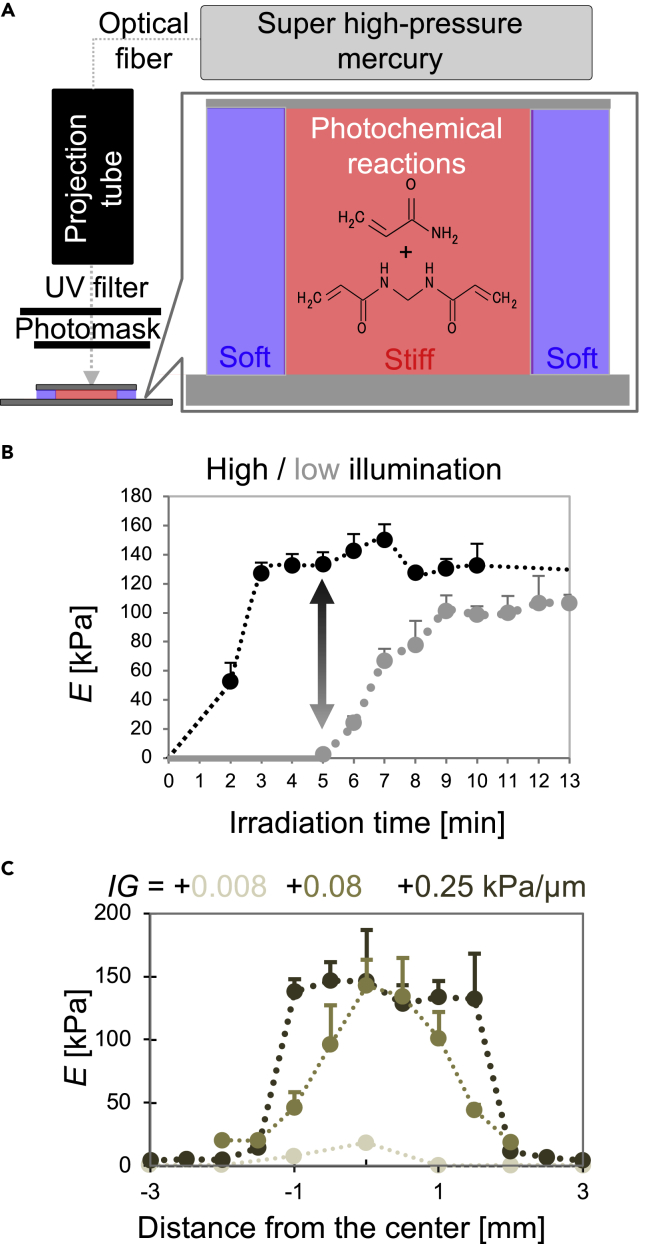
Preparation of hydrogel substrate with mechanical patterns (A) UV illumination system for the preparation of photoinitiated PAAm gels. (B) Time course of increased stiffness modulated by UV illumination time. (C) Mechanical distribution of locally stiffened PAAm gels with different mechanical gradients. The error bar represents the SD of Young’s modulus from n = 16 force curves in the square region (20 × 20 μm).

A hydrogel substrate with mechanical patterns was prepared with protocols for photo-initiated polyacrylamide (PAAm) gels (Tse and Engler, 2010). Briefly, a reaction solution was pre-irradiated with UV light (*λ*_*max*_ = 254 nm), and the following irradiation through a photomask (Figure 1A) leads to local stiffening. Since radicals produced by UV irradiation induce acrylamide polymerization, Young’s moduli of PAAm gels can be fine-tuned by adjusting UV-irradiation time (Figures 1B and 1C) and photomask patterns (Figure S1). Staining PAAm gels with a hydrophobic chromophore (trypan blue) clearly showed a center region with a higher crosslinking density than the surrounding region (Figure S1A, left). In this work, we first tested locally stiffened gel substrate with a mechanical gradient (slope of the intrinsic gradient around mechanical boundary: *IG* = +0.008, Figure 1C). From the comparison between low and high mechanical gradients for mesenchymal-driven self-condensation (Figure 4A), we finally prepared a PAAm gel with a stiff center region (*E*_*center*_ = 111.3 ± 10.9 kPa, diameter ∼ 4 mm) surrounded by a soft region (*E*_*surrounding*_ = 4.5 ± 2.6 kPa) with high mechanical gradient. The substrate showed the most prominent mechanical contrast, achieved by taking an optimal irradiation time (Figure 1B, double-headed arrow) for the liver organ bud formation. The *IG* of the substrate at the boundary between stiff and soft regions is ∼0.25 kPa/μm (125 kPa/500 μm). For cell adhesion, the PAAm gel surface was functionalized with a Matrigel layer, which is thin enough to induce cell mechano-response to the bulk mechanical properties of the underlying PAAm gel substrate (Takebe et al., 2015) (Buxboim et al., 2010). The details of the gel preparation and other experimental protocols are summarized in the STAR methods.

### Mesenchymal cell-driven self-condensation on the locally stiffened gel

**Figure 2:**
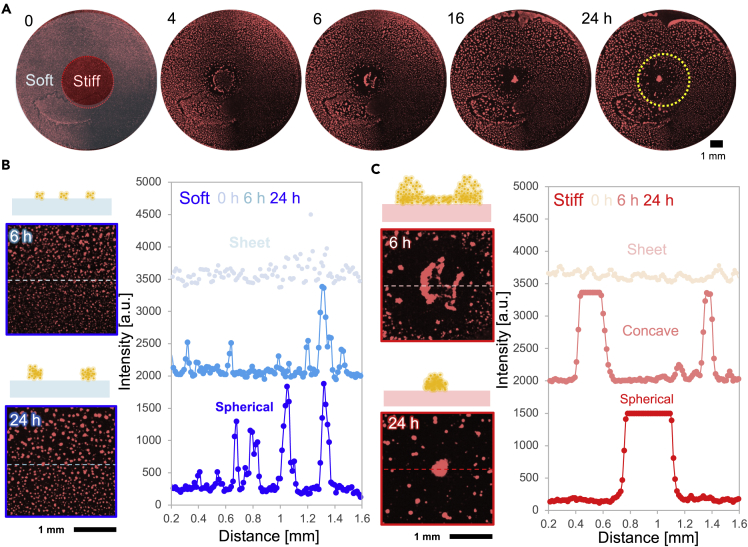
Local stiffening leads to symmetry breaks in the self-condensation and axial morphology (A–C) Time-lapse, fluorescence imaging of rat skeletal myoblast cellular condensation labeled with RFP-*F*-actin. For eye support, estimated mechanical boundaries are described with a yellow dotted circle. Magnification of fluorescence images of condensates and corresponding intensity profiles on (B) soft and (C) stiff regions.

For the first test of locally stiffened patterns, self-condensation and its dynamics of mesenchymal mammalian myoblast cell line (L6) with fluorescence *F*-actin networks were investigated. After plating cell mixtures (2×10^6^ cells/gel) on a locally stiffened hydrogel substrate, time-lapse fluorescence imaging was carried out (Figure 2A). At the early incubation stage (*t* ∼ 4h), a ring-like area without fluorescence signals was observed along the boundary between soft and stiff regions. Then we found the directed motion of cells on a stiffed region toward its center and it became a spherical condensate at around *t* = 24 h. The magnified images also showed its dynamics: concave-like condensates were first formed on the stiff region at *t* = 6 h, and the condensates showed concave to sphere morphological transition later (Figure 2C). On the other hand, on a soft region, a number of smaller spheroids (φ < 30 μm) were formed within *t* = 24 h (Figure 2B). To investigate the axial morphology of the condensates on soft/stiff regions, confocal images were obtained at *t* = 24 h (Figure S2A). Condensates on the soft region were spheroids with a thickness *h* ∼ 100 μm. In contrast, isolated condensates on the stiff region were not simple spheroid but complex morphology: large ellipsoidal condensates in the center with attaching fiber-like condensates (arrow). The maximum axial height of the condensate on a stiff region reaches ∼500 μm, which was not achieved on a soft region. Such self-condensation was driven by not epithelial but mesenchymal cells (Figure S2B). These results indicate that locally stiffened hydrogels lead to a symmetry break of mesenchymal condensates in the axial morphology, whereas mechanically homogeneous substrates cannot.

### Orchestration of adhesion and the intercellular cytoskeletal network defines two-step self-condensation

**Figure 3:**
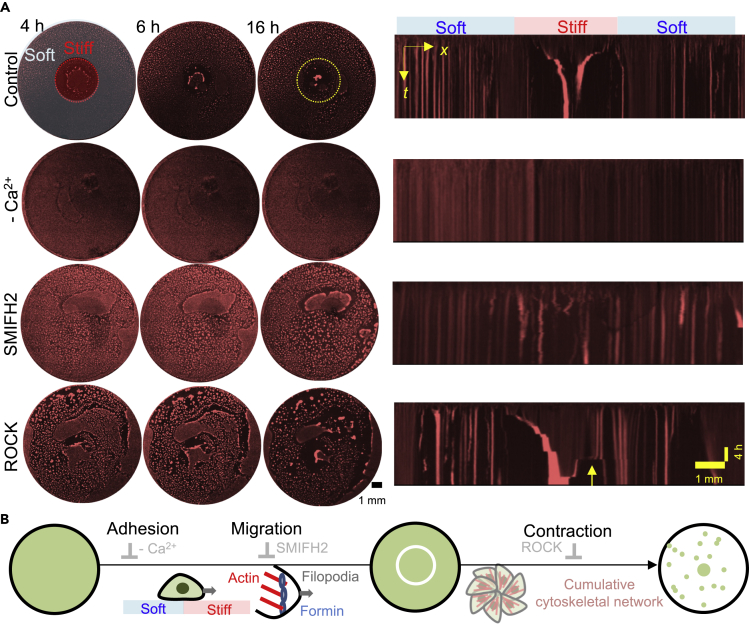
Inhibition of adhesion and cytoskeletal network suppress dynamic self-condensation on locally stiffened gels (A) Self-condensation dynamics in the absence and presence of inhibitors for cellular adhesion, migration, and contraction. Here, the right panels show corresponding time-lapse montage across the center of the gels. An estimated mechanical boundary is described with a yellow dotted circle for the eye support. (B) Proposed molecular dynamism for self-contraction on locally stiffened gels. Initial segregation at mechanical boundary driven by cellular migration.

To gain more physical insights into condensation dynamics, the time evolution of fluorescence intensity along a line across a center of the hydrogels was analyzed (Figure 3A, control). Mesenchymal condensates on a locally stiffened region showed two-step condensation dynamics (Figure S3A), which were also observed in the case of iPSC-based liver buds on a uniform Matrigel (Takebe et al., 2015). Primary condensation speed (*v*) was estimated to *v* ∼ 37 μm/h, which is comparable to the migration speed of mesenchymal fibroblast cells (Kuboki et al., 2019; Moriyama and Kidoaki, 2018). Here, the mechanical gradient (*IG* = 0.008 ka/μm) is comparable to the previously reported mechanical gradient to drive the directed motion of single smooth muscle cells (Choi et al., 2012; Whang and Kim, 2016; Wong et al., 2003; Zaari et al., 2004). Thus, mechanotaxis may play a critical role in the early stage of condensation. Afterwhile, condensates showed approximately 7 times faster movement (*v* ∼ 270 μm/h) (which was analyzed by line fitting from the trajectories in Figure S3A). In addition, the projection area of the condensate on a stiffened region became 80 times smaller. Such fast movement cannot be achieved by cell migration alone. Still, it should be attributed to the contraction of elastic bodies (*i.e.,* cell condensates in this study) (Geissler et al., 2009). In addition, our previous reports suggested that the formation of tissue-level cumulative cytoskeletal components such as stress fibers may trigger contraction. Thus, such two steps' condensation dynamics can be described by orchestrated primary cellular migration and secondary contraction of elastic cell condensates.

We used inhibitors for cell adhesion and intercellular cytoskeleton remodeling to answer the detailed molecular mechanism of dynamical contraction on locally stiffened gels. First, we focused on the role of cell adhesion (Figure 3A, -Ca^2+^). Addition of 1 mM EGTA (ethylene glycol tetra acetic acid) that chelates Ca^2+^ completely inhibited two steps of condensation: (1) primary cellular migration at the mechanical boundary and (2) secondary contraction on the stiff region. Depletion of Ca^2+^ results in the suppression of cell-cell adhesion via cell adhesion molecules (e.g., cadherin); thus, cell migration and cell contraction were completely suppressed. Addition of 1 mM EDTA for chelators of Mg^2+^ and Ca^2+^ completely inhibited contraction (Figure S3B top). Mg^2+^ has been considered to act as an essential player for cell-extracellular matrix (ECM) adhesion via integrin. Based on the obtained results, supported adhesion via cell adhesion molecules is one of the keys to self-condensation on the locally stiffened gels.

Next, we assessed the impact of inhibitors on cell migration onto self-condensation (Figure 3A middle). The addition of 10 μM SMIFH2 (specific inhibitor for formin, which is a stabilizing agent for filopodia) (Suraneni et al., 2015) significantly suppressed both cell migration and contraction. The addition of 10 nM paclitaxel, a representative inhibitor for ruffling and lamellipodium formation (Ballestrem et al., 2000; Choi and Yoo, 2012), also suppressed the self-condensation (Figure S3B middle). The obtained result highlighted the importance of primary segregation driven by cellular migration that triggers the subsequent elastic contraction process.

Finally, we assessed the impact of inhibitors on cell-cell contraction by regulating cytoskeleton remodeling. In the presence of inhibitors (100 μM) for Rho kinase (ROCK, regulator of cell-contraction via act-myosin activation (Liao et al., 2007; Toyoda et al., 2017)), cells failed the symmetric and clear condensation (Figure 3A, bottom right yellow arrow). Moreover, the addition of 10 μM blebbistatin, an inhibitor for non-muscle myosin heavy chain II A, also did not lead to complete inhibition of self-condensation, comparable to ROCK treatment cases: flattened condensates on the stiff region (Figures S3A and S3B bottom). Confocal fluorescence microscopy has several advantages in obtaining 1) dynamical insight into condensates (Figure 2A) and 2) fine topological profiles of condensates along the optical axis (Figures S3C–S3E). Another conventional microscopy cannot visualize such dynamical morphological change of condensates by blebbistatin with a deeper depth of field (*eg.*, stereomicroscope, scanning electron microscopy).

Based on these results, the initial segregation at the mechanical boundary supported by cell migration and the subsequent contraction process play critical roles in forming condensates on a stiffened region (Figure 3B). Adhesion of cell-ECM/cell-cell is essential for the early step of self-condensation. Then, cell migration via filopodia/lamellipodium drives the segregation at the mechanical boundaries. Such mechanotaxis ignited the subsequent contraction step, supported by cell-cell contraction force. Such mechano-boundary-dependent condensation will be helpful in controlling the formation of cell condensates spatially.

### Impact of locally stiffened pattern on mesenchymal cell-driven self-condensation

**Figure 4:**
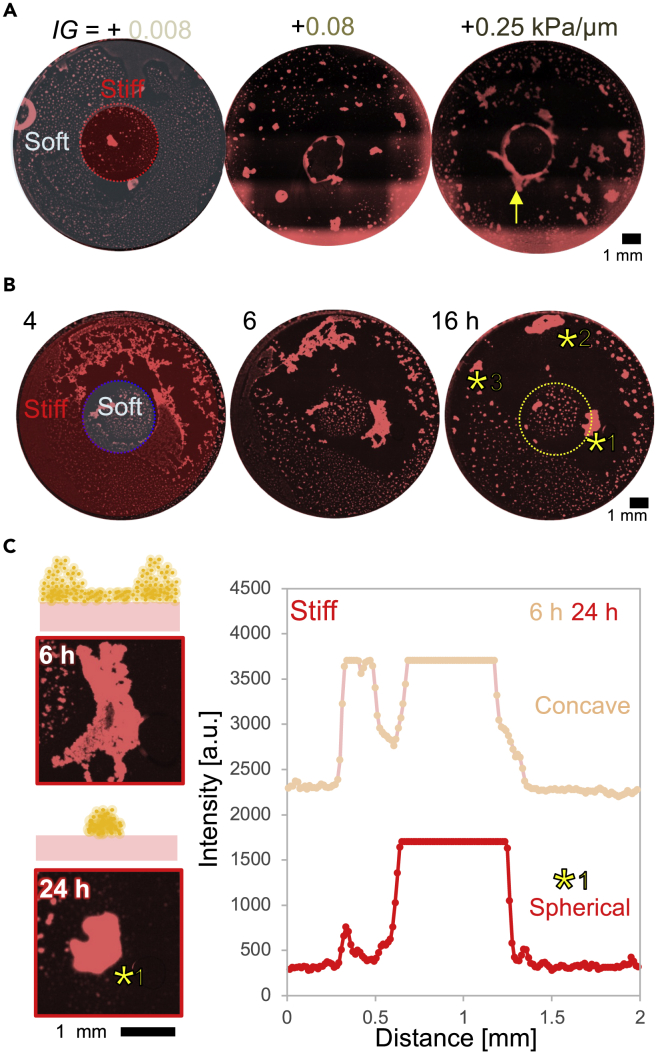
Mechanical pattern controls self-condensation (A) Impact of the magnitude of mechanical gradient onto self-condensation. Fluorescence images were obtained at day1. (B) Self-condensation on the locally softened gel. An estimated mechanical boundary is described with a yellow dotted circle for the eye support. (C) Magnification of spitted condensates (yellow asterisk) on the stiff region. Intensity profiles of condensates and the schematic illustration of condensation dynamics are displayed. Trends of splitting condensates were reproducible (n = 4, data not shown).

To validate the impact of the magnitude of mechanical gradient on self-condensation, we prepared locally stiffened gel with three different *IG* = +0.008, +0.08, and +0.25 (Figure 4A). Higher *IG* led to forming large condensates with ring-like structures at the boundary. The lateral and axial complexity of condensates can be enhanced with higher *IG*.

The next question is how inverted mechanical patterns (*i.e.*, a soft region surrounded by a stiff region) influence self-condensation. To answer this question, we prepared inverted patterns using a photomask. Although condensates were formed on a stiff region, the condensates were splitted (Figure 4B, asterisk). Such trends of splitting condensates were reproducible in four independent experiments (data not shown). Intensity profiles of the condensates supported the morphological transition of condensates on the stiff region from concave to spherical morphology (Figure 4C). Compared to the local stiffening pattern led to single isolated condensates on the stiff region (Figure 4A), the inverted pattern induced splitting of the condensates. According to previous *in vivo* studies, locally stiffening in the environment plays a crucial role in the morphogenesis of early and later embryos (Eiraku et al., 2011; Koser et al., 2016; Moore et al., 1995). Thus, these results highlight the impact of local stiffening patterns on the morphological development of “single” condensates.

### Preparatory self-condensation dynamics of liver organ buds on locally stiffened gels

**Figure 5:**
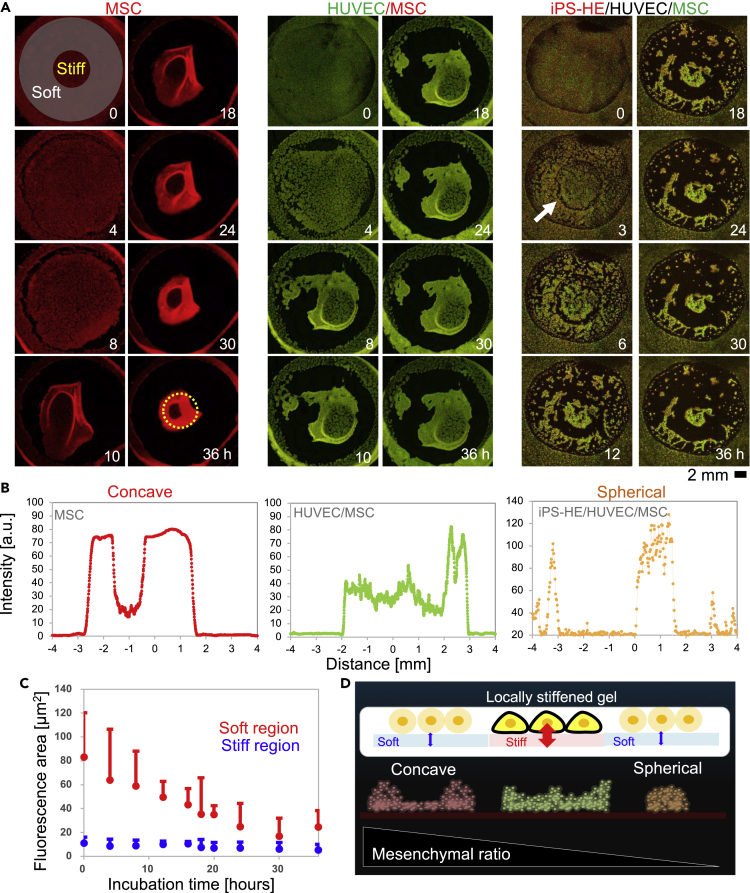
Impact of *in vivo*-inspired mechanical patterns on self-condensation of human liver buds (A and B) Time-lapse fluorescence imaging of condensation of MSC, MSC/HUVEC, and MSC/HUVEC/iPS-HE (liver buds) on the hydrogel with a mechanical pattern, and (B) the corresponding final morphology. An estimated mechanical boundary is described with a yellow dotted circle for the eye support. (C) Statistical analysis of fluorescence area on the stiff/soft region of the hydrogel with a mechanical pattern (n = 8). The soft region of the hydrogel is defined as the center of the hydrogel with a circle of 4 mm in diameter. (D) The proposed mechanism of controlling self-condensation morphology by the hydrogel with a mechanical distribution.

Figure 5A shows representative results of various cell condensation dynamics on the hydrogel substrate with the mechanical pattern. With MSC-only cultures, a two-dimensional cell sheet, the leading player for condensation via their contraction force (Takebe et al., 2015), was first formed and then started a drastic morphological transition from *t* ∼ 10 h (Figure 5A, MSC, Video S1). Cells in the surrounding soft region collectively moved toward the center (stiff region), while cells in the center region remained almost static. Next, a single condensate with concave morphology was formed at the center region (*t* = 36 h), which was confirmed by a fluorescence intensity profile of the formed condensate (Figure 5B, MSC). Notably, spherical cell condensates are typically formed with the use of homogeneous Matrigel substrate (Figure S5A) (Takebe et al., 2013). Coculturing MSCs and HUVECs, which conveys organoid vascularization (Takebe et al., 2013, 2015), also resulted in similar morphological dynamics; a two-dimensional sheet of MSCs/HUVECs moved toward the stiff region at the center (*t* = 6 h) (Figure 5A, HUVEC/MSC, Video S2). Then, a condensate with concave morphology formed on the central region, while the condensate was relatively wider and loosely packed (Figure 5B HUVEC) compared to that with MSCs alone. It should be noted that gels with a lower mechanical gradient (*IG* = 0.1) did not induce drastic self-condensation (Figure S5C). Thus, the higher mechanical gradient *IG* ∼ 0.25 is necessary for the self-condensation of the liver organ bud. Finally, we tested a total cell mixture for initial condensation of liver organ bud MSC/HUVEC/iPSC-HE. We found that a two-dimensional cell sheet showed clear separation at the boundary between stiff and soft regions (*t* = 6 h) (Figure 5A iPS-HE/HUVEC/MSC, Video S3). The intensity profiles of the image at *t* = 36 h revealed the formation of a large pancake-like condensate (*Φ* ∼ 2 mm, confocal data in Figure S5D) on the stiff region in contrast to many smaller island networks on the soft region (Figure 5B iPS-HE/HUVEC/MSC). These results indicated that complex cell mixtures of organ buds also experienced directed motion from soft to stiff regions on the mechanically patterned gel. The decrease in MSC content supported the morphological transition of condensates from concave to concave to pancakes (Figure 5C).

Video S1. Condensation of MS on mechanically patterned hydrogel, related to Figure 5A left

Video S2. Condensation of HUVEC/MSC on mechanically patterned hydrogel, related to Figure 5A middle

Video S3. Condensation of iPS-HE/HUVEC/MSC on mechanically patterned hydrogel, related to Figure 5A

## Discussions

These results indicate that our hydrogel substrates can provide symmetry breaking in cell condensates according to mechanical patterns. As shown in Figure 5A, cell movement was different between stiff and soft regions. The time course of the hydrogel area covered by MSC/HUVEC mixtures during incubation (*t* = 36 h) statistically showed that cells in the soft region were dynamic (83 ± 37 μm^2^ to 17 ± 15 μm^2^, n = 8), while cells on the stiff region remained almost static (11 ± 5 μm^2^ to 5 ± 5 μm^2^, n = 8) (Figure 5C). The underlying mechanism of this symmetry breaking in the morphologies of cell condensates can probably be explained by cell-cell (*F*_*cell-cell*_) and cell-hydrogel (*F*_*cell-gel*_) mechanical interactions. Our recent studies revealed that cell condensation is driven by the mechano-sensitive contraction and adhesion force of MSCs. If gel substrates are moderately soft (*e.g.,* Matrigel), spherical condensates are typically formed (Figure S5A) because MSCs show weaker adhesion to softer substrates (Yoshikawa et al., 2011); thus, cell-cell contraction and adhesion forces can defeat cell-gel adhesion force (*i.e., F*_*cell-cell*_ > *F*_*cell-gel*_) (Shinozawa et al., 2016). Therefore, hydrogel substrates with mechanical patterns should cause different force situations between stiff and soft regions, forming cell condensates with non-spherical shapes (Figure 5D). For example, concave morphology (Figure 5A MSC) should appear because MSCs form a 2D sheet on a stiff region due to strong cell-gel adhesion, while MSCs are very mobile on a soft region. The formation of a wider and loosely packed condensate by MSCs/HUVECs (Figure 5A HUVEC) is attributed to a decrease in the MSC ratio in the cell mixture, which should result in weaker cell-cell contraction adhesion forces during condensation. In addition, the formation of spherical condensate by MSC/HUVEC/iPS-HE mixtures can be explained by weakening *F*_*cell-cell*_ compared to *F*_*cell-gel*_. It should also be noted that the intrinsic stiffness gradient at the boundary between stiff and soft regions (*IG* ∼ 0.25 kPa/μm) is lower than the threshold stiffness gradient (*TG* ∼ 1.0 kPa/μm) for durotaxis of MSCs on a mechanically patterned hydrogel where the stiffness of soft region is 5 kPa (Moriyama and Kidoaki, 2018). In addition, the maximum velocity of the movement of the MSCs condensate edge shown in Figure S5B (∼1200 μm/h) was much higher than that reported for cell migration (Dourdin et al., 2001; Wolf et al., 2003). These facts also support that the pattern formation of MSC condensate is driven not by active cell migration but by mechano-sensitive cell contraction. In contrast, cell separation at the mechanical boundary was visualized according to a decrease in MSC ratio (Figure 5A iPSC-HE/HUVEC/MSC, white arrows). The results indicate that self-condensation of liver organ bud was also two-step contractions: primary cellular migration at the mechanical boundary and secondary self-contraction via cell-cell contraction.

According to recent *in vivo* studies, local stiffening of extracellular environments during developmental processes is essential in inducing symmetry breaking in tissue morphology. For example, the initial symmetry break in Xenopus embryos is ignited by the locally stiffening region, which leads to the archenteron with concave morphology (Moore et al., 1995). Interestingly, the magnitude of local stiffening (*i.e.,* the ratio of elasticity between hard and soft ratio, the H/S ratio) used in this study (*H/S* = 24) is comparable to that of Xenopus embryos (*H/S* = 2–20) during crucial stages of neural tube closure (stage 11.5–21) (Zhou et al., 2009). Moreover, morphological transition in the latter developmental process (*i.e.*, optical cup formation) is supported by the surrounding stiffened retinal pigment epithelium (*H/S* = 2–16), driving cellular inward movement (Eiraku et al., 2011). Here, a mechanical gradient with low contrast did not induce cellular migration at the mechanical boundary and targeted contraction on the stiff region (Figure S5C). Considering our results and these facts obtained from *in vivo* studies, locally stiffened hydrogels can offer biologically relevant mechanical environments that direct self-condensation with asymmetric morphology, promoting the early and later developmental process of organoids.

### Limitations of the study

In conclusion, we investigated spatial mechanical patterns' impact on the morphologies of the initial self-condensation of differentiation progeny (from myoblast to liver organ buds and their constituents). Benefiting from the *in vivo*-inspired mechanical pattern on PAAm hydrogels, symmetrical breaks in condensates can be spatially controlled using mechanically patterned hydrogel substrates. Morphology of condensates was also regulated by the underlying mechanical pattern (*i.e.,* small to large circular, triangle, and star: Figure S4). The underlying mechanics can be explained by physical modeling based on force competition between *F*_*cell-gel*_ and *F*_*cell-cell*_. Moreover, the underlying molecular mechanism of self-contraction is supported by primary cell migration and secondary cell contraction. Although the impact of mechanical patterns on the maturation of condensates is currently limited and will be discussed in the next coming papers, previous studies have already indicated that spherical liver organ buds express liver function after transplantation (Takebe et al., 2013). Comparable size and reproducibility of internal segregation of cells (Asai et al., 2017) were reproducible (Figure S5D right). Moreover, long cultivation of condensates induced elongated shapes of myoblast inside condensates (Figures S2C and S2D), supporting the presence of myogenic differentiation. Thus, we believe that the *in vivo-*inspired design of culturing platforms with mechanical patterns will lead to breakthroughs for further development of organoids with asymmetric morphologies for future clinical application (Takebe et al., 2017).

## STAR★Methods

### Key resources table

**Table:** 

REAGENT or RESOURCE	SOURCE	IDENTIFIER
**Chemicals, peptides, and recombinant proteins**		

Deionized water	Advantech	GS-200
MilliQ-Synergy	Merk	SYNS0HFWW
circular glass slip	Matsunami	C012001
Acetone	Wako	019-00353
Methanol	Wako	137-01823
Ethanol	Wako	057-00451
Chloroform	Wako	038-02601
allyltrichlorosilane	Merk	107778-5G
40 %v/v Acrylamide	Merk	A4058
2% v/v N,N′-Methylenebisacrylamide	Merk	M1533
Irgacure 2959 (Omnirad 2959)	IGM Resins B.V.	N/A
dichlorodimethylsilane	Merk	40140
acetylcellulose film	Agar Scientific Ltd,	G254B
Matrigel Basement Menbrane10 mL	Corning	354234
sulfosuccinimidyl-6-[4′-azido-2′-nitrophenylamino]hexanoate	Thermo	22589
HEPES(4-(2-hydroxyethyl)-1-piperazineethanesulfonic acid)	Dojino	346-01373
RPMI 1640	Nissui	05911
penicillin–streptomycin	Wako	168-23191
non-essential amino acid	Wako	139-15651
Glutamax	Thermo	35050-061
Sodium bicarbonate	Thermo	25080-094
MSCGM	Lonza	PT-3001
EGM	Lonza	CC-3124
HCM	Lonza	CC-3198

**Software and algorithms**		

Igor 6.3 Pro	Wavemetrix	https://www.wavemetrics.com/order/order_igordownloads6.htm
JPK data processing	Bruker	https://japan.jpk.com/downloads
Nis-ElementsC	Nikon	https://www.microscope.healthcare.nikon.com/ja_JP/products/software/nis-elements

**Other**		

Laser printer	OKI	MC860
illuminometer	Konica Minolta Inc.	T-10A,
Atomic force microscopy (AFM)	JPK	Nanowizard3
UV lamp	Sigma	Z169633-1EA
Cantilever	Olympus	OMCL-RC800-PSA
Tokai hit incubator	Tokai hit	INU8H-ZILCS-F1
Confocal microscopy	Nikon/Leica	C2 or A1HDR25/SP8

### Resource availability

#### Lead contact

Further information and requests for resources, measurement procedures, and data can be directed to the lead contact, Dr. Takahisa Matsuzaki (matsuzaki@ap.eng.osaka-u.ac.jp).

#### Materials availability

This study did not generate new unique reagents.

### Experimental model and subject details

Myoblast from rat skeletal muscle (L6, CRL-1458, ATCC) was spread onto mechanically patterned gels in 24 well glass plate For the condensation of cells, RPMI 1640 Medium (Nissui) supplemented with 10% v/v fetal bovine serum (FBS, Invitrogen, Tokyo, Japan) and 1% v/v penicillin–streptomycin solution (PS, Wako Pure Chemicals, Osaka, Japan), non-essential aminnoacid, Glutamax and 0.2% v/v sodium carbonate were used in a Tokai hit incubator (5% CO2, 37°C). For the fluorescence imaging of L6 condensation, we established stable cell lines expressing the F-actin reporter LifeAct by retrovirus infection. First, LifeAct peptide (MGVADLIKKFESISKEE) coding sequence fused with monomeric RFP (mRFP) at its C-terminus was cloned into pMXs-puro. PLAT-E cells cultured for retrovirus packaging and retrovirus production (Yoneyama et al., 2018). L6 cells were incubated with a virus-containing medium supplemented with 2 μg/mL polybrene. Then uninfected cells were removed by puromycin selection.

Undifferentiated human iPSCs (TKDA3-4) were maintained without feeder cells in iPSC cell medium. Human iPS-HE, human bone marrow-derived MSCs (Lonza), and HUVECs (Lonza) were cultured hepatocyte cultured medium, mesenchymal cultured medium (MSCGM, Lonza, Tokyo, Japan), and endothelial growth medium(Takebe et al., 2013, 2015) For cell condensation on the substrate, cell mixtures were resuspended in a mixture of endothelial growth medium (EGM, Lonza, Tokyo, Japan) and hepatocyte culture medium (HCM, Cambrex, NJ, USA) containing dexamethasone (0.1 mM, Sigm), oncostatin M (10 ng/mL, R&D Systems, NE, USA), hepatocyte growth factor (HGF, 20 ng/mL, Kringle Pharma, Inc., Osaka, Japan), and SingleQuots (EGM, Lonza, Tokyo, Japan). Four milliliters cell suspension was plated onto presolidified Matrigel (BD Biosciences, Tokyo, Japan) or mechanically patterned PAAm films in a 24-well plate and maintained at 37°C with 5% CO_2_ by using an incubation chamber (TokaiHit, Shizuoka, Japan) on an inverted microscope. Here, the combination of the ratio of cells was iPSC-HE: HUVEC: MSC = 2: 4: 5, HUVC: MSC = 1: 4, and MSC = 1, which corresponds to percentages of MSC = 46, 80, and 100%, respectively. The total number of cells spread on the substrate was fixed at 2×10^6^.

### Method details

#### Materials and reagents

Deionized water from a Milli-Q device (Japan Millipore Ltd, Tokyo, Japan) was used throughout this study. Unless stated otherwise, all other chemicals were purchased from Wako (Tokyo, Japan) or Life Technologies (Tokyo, Japan) and used without further purification.

#### Preparation of mechanically patterned PAAm gel films

Mechanically patterned polyacrylamide (PAAm) gels were prepared on aminosilanized glass coverslips by improving previous protocols which were proposed by the previous researchers(Buxboim et al., 2010; Tse and Engler, 2010). In brief, as a supporting glass substrate, round glass slips (Φ = 12 mm in diameter, thickness = 0.12-0.17, Matsunami, Tokyo, Japan) were cleaned using a modified RCA method (Kern, 1970) and then functionalized using allyltrichlorosilane (ATCS, Sigma, Tokyo, Japan). For the preparation of precursor solution, 3 mL acrylamide solution (40% Sigma), 3 mL bis-acrylamide (2% Sigma), 4 mL distilled water, and 50 mg Irgacure 2959 (BASF Inc., Tokyo, Japan) were mixed under a 37°C water bath until the powder dissolved. After degassing the solution under vacuum, 12 μL degassed solution was deposited onto a dichlorodimethylsilane (DCDMS, TCL, Sigma)-treated hydrophobic slide glass, and the aminosilated coverslip was placed on top. To obtain spatial mechanical distribution on the hydrogel, we projected patterned UV light onto the precursor solution using a custom-made illumination device (Figure 1A). For the preparation of a photomask, a black ink pattern was printed onto acetylcellulose film (G254B, Agar Scientific Ltd, Essex, UK) by using a conventional printer (OKI, MC860, Tokyo, Japan). Photomasks used in this study to achieve mechanical distribution on a PAAm gel film in the stiff circular region in the center are shown in Figure 1C. The stiffness of PAAm was finely tuned by the UV irradiation time through the mask with or without black ink on the mask, which enabled us to change the stiffness by a factor of 50 (E = 2–100 kPa). The illuminated UV intensity was ∼4.9 mW/cm^2^, as measured by a conventional illuminometer (T-10A, Konica Minolta Inc., Tokyo, Japan). To obtain the mechanical distribution on the PAAm gel film, we pre-illuminated the precursor solution without a mask for 1 min and 7 min with a positive patterned mask. For cell adhesion, Matrigel was chemically cross-linked with the PAAm surface according to our previous protocols(Tse and Engler, 2010). In brief, 300 μL 0.2 mg/mL sulfosuccinimidyl-6-[4′-azido-2′-nitrophenylamino]hexanoate (sulfo-SANPAH, Thermo Fischer Scientific, Yokohama, Japan) in 20 mM HEPES (pH = 6.8) was pipetted onto the entire surface of the hydrogel. Then, the hydrogel was illuminated using a UV lamp (Z169633-1EA, Sigma) for 20 min. Finally, 300 μL Matrigel solution (4.4 μg/mL in final) was deposited onto the gel and incubated overnight at 37°C. The prepared gel substrate was stored at 4°C until use.

#### Nanoindentation of mechanically patterned PAAm gel films

Nanoindentation of hydrogel was performed using an atomic force microscope (AFM, Nano Wizard 3, JPK instruments, Berlin, Germany). Commercial silicon cantilevers with spring constant k = 0.76 N/m with a pyramidal tip (OMCL-RC800-PSA, Olympus, Tokyo, Japan) were used. Force−indentation (f−i) curves of hydrogel were obtained in 10 mM Dulbecco’s phosphate buffered saline solution (pH = 7.4). Apparent Young’s modulus, E, of the hydrogels was calculated from the approach curves using Sneddon’s modification of the Hertz model. (Hertz, 1881; Sneddon, 1965)

(Equation 1) $$F=\;\frac{2tan\left(\alpha \right)}{\pi }\frac{E}{1-\mu^{2}}\delta^{2}\;$$

where F is the load, δ is the indentation depth, μ is the Poisson ratio, and α is a semi-vertical angle of the indenter. In this study, μ and α were assumed to be 0.45 and 35°, respectively. The maximum indentation depth was set to 2 μm, and E was obtained by fitting the region from 0-2 μm.

## Data Availability

• Data reported in this paper will be shared by the lead contact upon reasonable request. • This paper does not report original code. • Any additional information required to reanalyze the data reported in this paper is available from the lead contact upon request upon reasonable request.

## References

[bib1] Asai A., Aihara E., Watson C., Mourya R., Mizuochi T., Shivakumar P., Phelan K., Mayhew C., Helmrath M., Takebe T. (2017). Paracrine signals regulate human liver organoid maturation from induced pluripotent stem cells. Development.

[bib2] Ballestrem C., Wehrle-Haller B., Hinz B., Imhof B.A. (2000). Actin-dependent lamellipodia formation and microtubule-dependent tail retraction control-directed cell migration. Mol. Biol. Cell.

[bib3] Buxboim A., Rajagopal K., Brown A.E.X., Discher D.E. (2010). How deeply cells feel: methods for thin gels. J. Phys. Condens. Matter.

[bib4] Choi Y.H., Yoo Y.H. (2012). Taxol-induced growth arrest and apoptosis is associated with the upregulation of the Cdk inhibitor, p21WAF1/CIP1, in human breast cancer cells. Oncol. Rep..

[bib5] Choi Y.S., Vincent L.G., Lee A.R., Kretchmer K.C., Chirasatitsin S., Dobke M.K., Engler A.J. (2012). The alignment and fusion assembly of adipose-derived stem cells on mechanically patterned matrices. Biomaterials.

[bib6] Discher D.E., Janmey P., Wang Y.-l. (2005). Tissue cells feel and respond to the stiffness of their substrate. Science.

[bib7] Dourdin N., Bhatt A.K., Dutt P., Greer P.A., Arthur J.S., Elce J.S., Huttenlocher A. (2001). Reduced cell migration and disruption of the actin cytoskeleton in calpain-deficient embryonic fibroblasts. J. Biol. Chem..

[bib8] Eiraku M., Takata N., Ishibashi H., Kawada M., Sakakura E., Okuda S., Sekiguchi K., Adachi T., Sasai Y. (2011). Self-organizing optic-cup morphogenesis in three-dimensional culture. Nature.

[bib9] Engler A.J., Sen S., Sweeney H.L., Discher D.E. (2006). Matrix elasticity directs stem cell lineage specification. Cell.

[bib10] Geissler M., Roy E., Deneault J.S., Arbour M., Diaz-Quijada G.A., Nantel A., Veres T. (2009). Stretching the stamp: a flexible approach to the fabrication of miniaturized DNA arrays. Small.

[bib11] Haraguchi Y., Shimizu T., Sasagawa T., Sekine H., Sakaguchi K., Kikuchi T., Sekine W., Sekiya S., Yamato M., Umezu M. (2012). Fabrication of functional three-dimensional tissues by stacking cell sheets in vitro. Nat. Protoc..

[bib12] Hertz H. (1881). On the contact of elastic solids. J. für die Reine Angewandte Math. (Crelle's J.).

[bib13] Kern W. (1970). Cleaning Solution-based on hydrogen peroxide for use in silicon semiconductor technology. RCA Rev..

[bib14] Koser D.E., Thompson A.J., Foster S.K., Dwivedy A., Pillai E.K., Sheridan G.K., Svoboda H., Viana M., Costa L.d.F., Guck J. (2016). Mechanosensing is critical for axon growth in the developing brain. Nat. Neurosci..

[bib15] Kuboki T., Ebata H., Matsuda T., Arai Y., Nagai T., Kidoaki S. (2019). Hierarchical development of motile polarity in durotactic cells just crossing an elasticity boundary. Cell Struct. Funct..

[bib16] Lancaster M.A., Knoblich J.A. (2014). Organogenesis in a dish: modeling development and disease using organoid technologies. Science.

[bib17] Liao J.K., Seto M., Noma K. (2007). Rho kinase (ROCK) inhibitors. J. Cardiovasc. Pharmacol..

[bib18] Matsumoto K., Yoshitomi H., Rossant J., Zaret K.S. (2001). Liver organogenesis promoted by endothelial cells prior to vascular function. Science.

[bib19] Matsusaki M., Sakaue K., Kadowaki K., Akashi M. (2013). Three-dimensional human tissue chips fabricated by rapid and automatic inkjet cell printing. Adv. Healthc. Mater..

[bib20] Moore S.W., Keller R.E., Koehl M.A. (1995). The dorsal involuting marginal zone stiffens anisotropically during its convergent extension in the gastrula of Xenopus laevis. Development.

[bib21] Moriyama K., Kidoaki S. (2018). Cellular durotaxis revisited: initial-position-dependent determination of the threshold stiffness gradient to induce durotaxis. Langmuir.

[bib22] Sasai Y. (2013). Cytosystems dynamics in self-organization of tissue architecture. Nature.

[bib23] Shinozawa T., Yoshikawa H.Y., Takebe T. (2016). Reverse engineering liver buds through self-driven condensation and organization towards medical application. Dev. Biol..

[bib24] Simunovic M., Brivanlou A.H. (2017). Embryoids, organoids and gastruloids: new approaches to understanding embryogenesis. Development.

[bib25] Sneddon I.N. (1965). The relation between load and penetration in the axisymmetric Boussinesq problem for a punch of arbitrary profile. Int. J. Eng. Sci..

[bib26] Suraneni P., Fogelson B., Rubinstein B., Noguera P., Volkmann N., Hanein D., Mogilner A., Li R. (2015). A mechanism of leading-edge protrusion in the absence of Arp2/3 complex. Mol. Biol. Cell.

[bib27] Takebe T., Enomura M., Yoshizawa E., Kimura M., Koike H., Ueno Y., Matsuzaki T., Yamazaki T., Toyohara T., Osafune K. (2015). Vascularized and complex organ buds from diverse tissues via mesenchymal cell-driven condensation. Cell Stem Cell.

[bib28] Takebe T., Sekine K., Enomura M., Koike H., Kimura M., Ogaeri T., Zhang R.R., Ueno Y., Zheng Y.W., Koike N. (2013). Vascularized and functional human liver from an iPSC-derived organ bud transplant. Nature.

[bib29] Takebe T., Sekine K., Kimura M., Yoshizawa E., Ayano S., Koido M., Funayama S., Nakanishi N., Hisai T., Kobayashi T. (2017). Massive and reproducible production of liver buds entirely from human pluripotent stem cells. Cell Rep..

[bib30] Toyoda T., Kimura A., Tanaka H., Ameku T., Mima A., Hirose Y., Nakamura M., Watanabe A., Osafune K. (2017). Rho-associated kinases and non-muscle myosin IIs inhibit the differentiation of human iPSCs to pancreatic endoderm. Stem Cell Rep..

[bib31] Tse J.R., Engler A.J. (2010). Preparation of hydrogel substrates with tunable mechanical properties. Curr. Protoc. Cell Biol..

[bib32] Whang M., Kim J. (2016). Synthetic hydrogels with stiffness gradients for durotaxis study and tissue engineering scaffolds. Tissue Eng. Regen. Med..

[bib33] Wolf K., Mazo I., Leung H., Engelke K., von Andrian U.H., Deryugina E.I., Strongin A.Y., Bröcker E.B., Friedl P. (2003). Compensation mechanism in tumor cell migration. J. Cell Biol..

[bib34] Wong J.Y., Velasco A., Rajagopalan P., Pham Q. (2003). Directed movement of vascular smooth muscle cells on gradient-compliant hydrogels. Langmuir.

[bib35] Yin X., Mead B.E., Safaee H., Langer R., Karp J.M., Levy O. (2016). Engineering stem cell organoids. Cell Stem Cell.

[bib36] Yoneyama Y., Lanzerstorfer P., Niwa H., Umehara T., Shibano T., Yokoyama S., Chida K., Weghuber J., Hakuno F., Takahashi S.I. (2018). IRS-1 acts as an endocytic regulator of IGF-I receptor to facilitate sustained IGF signaling. Elife.

[bib37] Yoshikawa H.Y., Rossetti F.F., Kaufmann S., Kaindl T., Madsen J., Engel U., Lewis A.L., Armes S.P., Tanaka M. (2011). Quantitative evaluation of mechanosensing of cells on dynamically tunable hydrogels. J. Am. Chem. Soc..

[bib38] Zaari N., Rajagopalan P., Kim S.K., Engler A.J., Wong J.Y. (2004). Photopolymerization in microfluidic gradient generators: microscale control of substrate compliance to manipulate cell response. Adv. Mater..

[bib39] Zhou J., Kim H.Y., Davidson L.A. (2009). Actomyosin stiffens the vertebrate embryo during crucial stages of elongation and neural tube closure. Development.

