# Supplementary material for: Mechanical guidance of self-condensation patterns of differentiating progeny
Source: iScience. 2022 Sep 27;25(10):105109. doi: 10.1016/j.isci.2022.105109 (PMC9617469; doi:10.1016/j.isci.2022.105109)
Supplement: Document S1. Figures S1–S5 [file mmc1.pdf]

iScience, Volume *25*

Supplemental information

Mechanical guidance of self-condensation patterns of differentiating progeny

Takahisa Matsuzaki, Yuko Shimokawa, Hiroyuki Koike, Masaki Kimura, Yuma Kawano, Nao Okuma, Ryuzo Kawamura, Yosuke Yoneyama, Yasuro Furuichi, Fumihiko Hakuno, Shin-Ichiro Takahashi, Seiichiro Nakabayashi, Satoshi Okamoto, Hiromitsu Nakauchi, Hideki Taniguchi, Takanori Takebe, and Hiroshi Y. Yoshikawa

Supporting information:

Mechanical guidance of self-condensation patterns of differentiating progeny

Takahisa Matsuzaki[1,2,3,4*], Yuko Shimokawa[4], Hiroyuki Koike[5,6], Masaki Kimura[5,6], Yuma Kawano[4], Nao Okuma[4], Ryuzo Kawamura[4], Yosuke Yoneyama[7,8], Yasuro Furuichi[9], Fumihiko Hakuno[8], Shin-Ichiro Takahashi[8], Seiichiro Nakabayashi[3,4], Satoshi Okamoto[10,11], Hiromitsu Nakauchi[12,13], Hideki Taniguchi[10,11], Takanori Takebe,[5,6,7*] and Hiroshi Y. Yoshikawa[2,3,4*]

[1]Center for Future Innovation, Graduate school of engineering, Osaka University, Suita, Osaka 565-0871, Japan.
[2]Department of Applied Physics, Graduate School of Engineering, Osaka University, Suita, Osaka 565-0871, Japan
[3]Division of Strategic Research and Development, Graduate School of Science and Engineering, Saitama University, Shimo-Okubo 255, Sakura-Ku, Saitama 338-8570, Japan.
[4]Department of Chemistry, Saitama University, Shimo-okubo 255, Sakura-ku, Saitama 338-8570, Japan.
[5]Department of Pediatrics, University of Cincinnati College of Medicine, 3333 Burnet Avenue, Cincinnati, OH, 45229- 3039, USA.
[6]Division of Gastroenterology, Hepatology & Nutrition, Developmental Biology, Center for Stem Cell and Organoid Medicine (CuSTOM), Cincinnati Children's Hospital Medical Center, 3333 Burnet Avenue, Cincinnati, OH 45229-3039, USA.
[7]Institute of Research, Division of Advanced Multidisciplinary Research, Tokyo Medical and Dental University, 1-5-45 Yushima, Bunkyo-Ku, Tokyo 113-8510, Japan.
[8]Departments of Animal Sciences and Applied Biological Chemistry, Graduate School of Agriculture and Life Sciences, The University of Tokyo, Bunkyo-Ku, Tokyo 113-8657, Japan.
[9]Department of Health Promotion Sciences, Graduate School of Human Health Sciences, Tokyo Metropolitan University, 1-1 Minami-Osawa, Hachioji, Tokyo, Japan.
[10] Department of Regenerative Medicine, Graduate School of Medicine, Yokohama City University, Kanazawa-ku 3-9, Yokohama, Kanagawa, 236-0004, Japan.
[11] Division of Regenerative Medicine, University of Tokyo, 4-6-1, Shirokanedai, Minato-Ku, Tokyo, 108-8639, Japan
[12]Institute for Stem Cell Biology and Regenerative Medicine, School of Medicine, Stanford University, Stanford, CA 94305, USA.
[13]Center for Stem Cell Biology and Regenerative Medicine, Institute of Medical Science, University of Tokyo, Minato-Ku, Tokyo, Japan.

[*]Triple corresponding authors.

Takahisa Matsuzaki
mail: matsuzaki@ap.eng.osaka-u.ac.jp Tel: +81-6-6879-7838

Hiroshi Y Yoshikawa
mail: hiroshi@ap.eng.osaka-u.ac.jpTel: +81-6-6879-7838

Takanori Takebe
mail: Takanori.Takebe@cchmc.org Tel: 513-803-7807

1. <u>Supporting Figures</u>

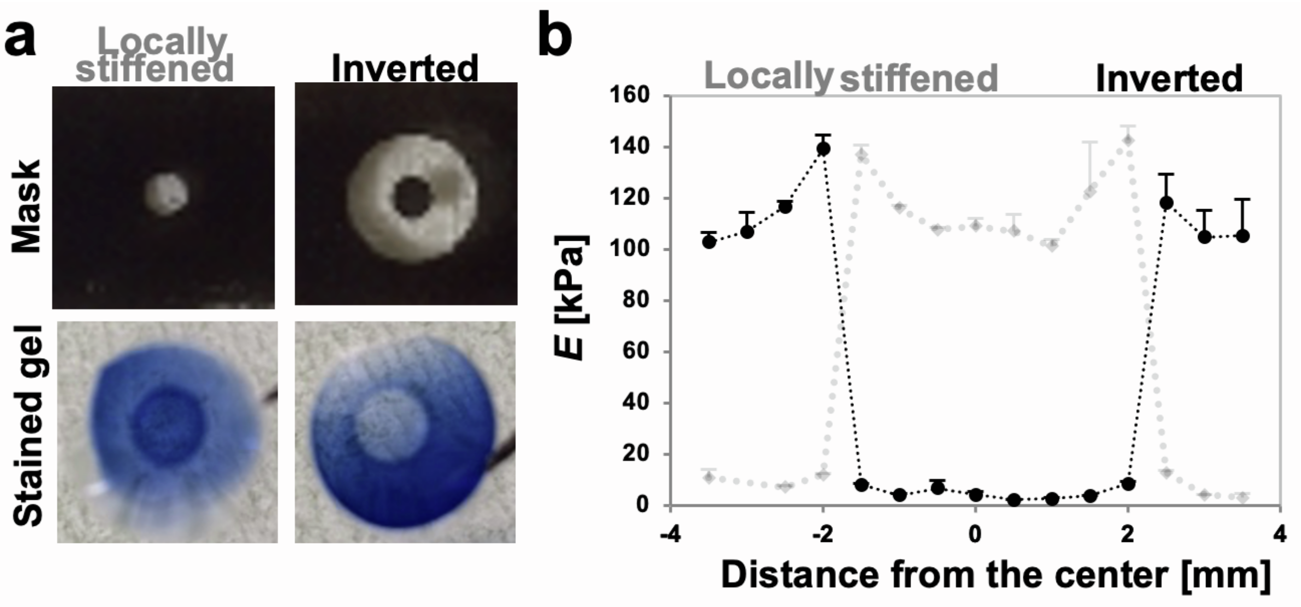

Figure S1: Physical characterization of mechanically patterned gels (Related to Figure 1). (a) Wide-filed image of photomask and prepared patterned gels stained with trypan blue. (b) Line profile of Young's modulus across the center of gels for locally stiffened and softened gels. Mean Young's modulus was characterized by atomic force microscopy (AFM, JPK instruments). The error bar represents the standard deviation of Young's modulus from n = 16 force curves in the square region (20 μm×20μm).

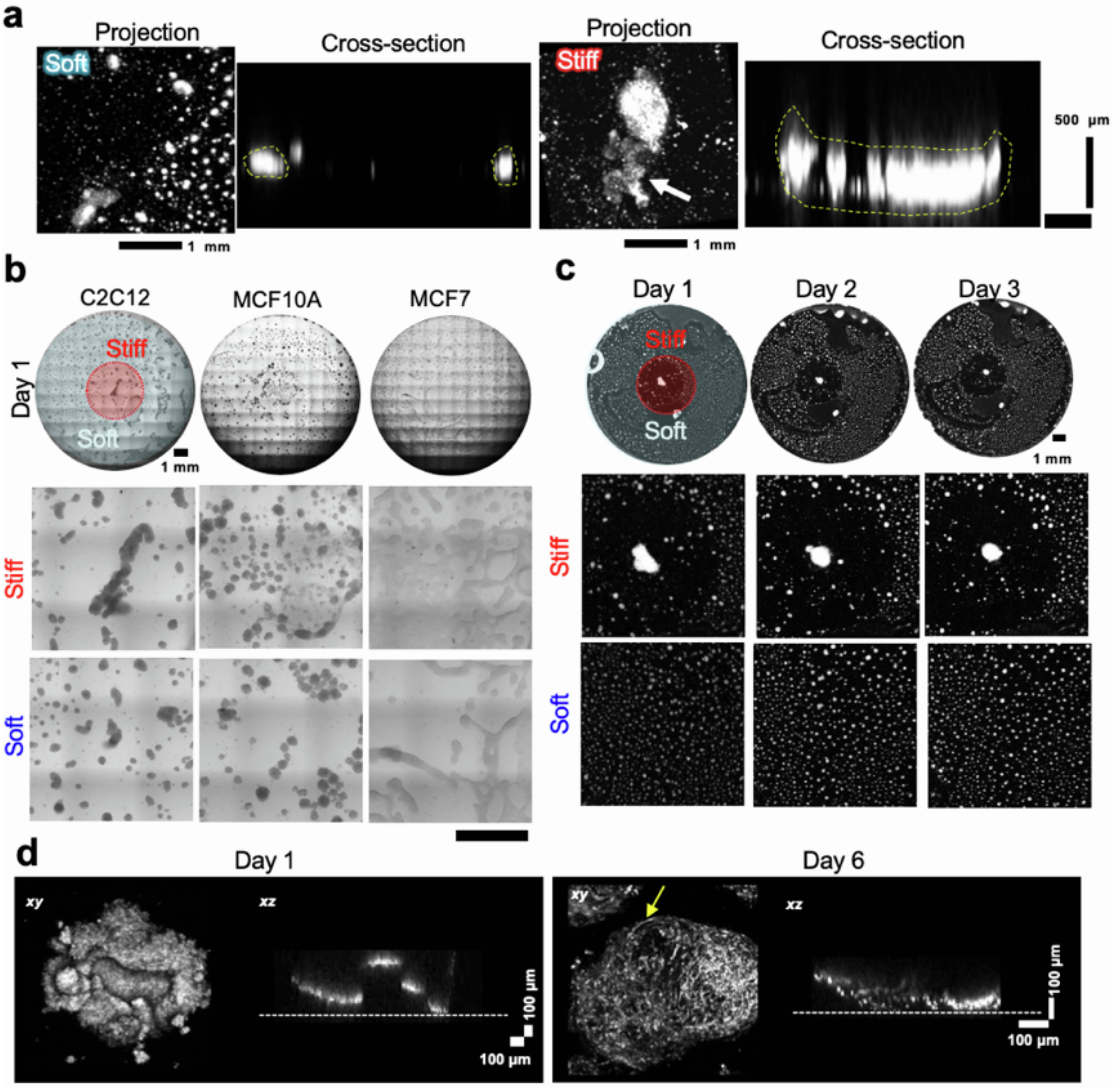

Figure S2: Morphological characterization of condensates, epithelial and mesenchymal cell-driven self-condensation, and its maturation (Related to Figure 2). (a)Validation of cellular types on self-condensation. Human epithelial breast cell lines (MCF10A and MCF7) were used. Epithelial cells did not induce single condensates onto stiff regions compared to mesenchymal mouse myoblast (C2C12). (c) Long cultivation of myoblast condensates on the mechanical pattern. For the differentiation of myoblast, we exchanged the medium with low content of house serum (2 %). (d) Representative large myoblast condensates are prepared by multiple mechanical patterns (data for day 1 is redisplayed in Figure S4c) and the myogenic differentiation. Confocal fluorescence microscopy detects the elongated cells (a yellow arrow) inside condensates, indicating the presence of myogenic differentiation.

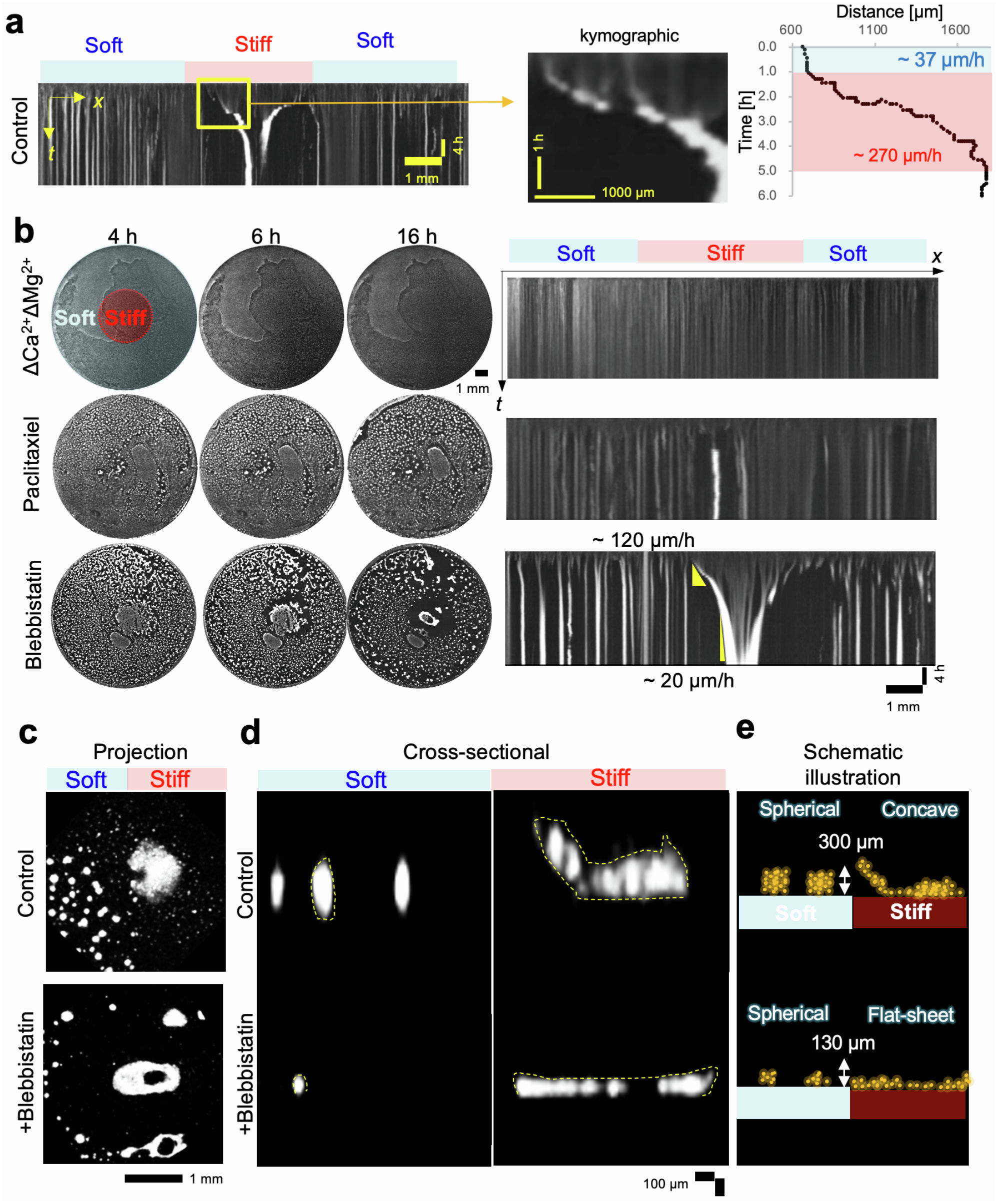

Figure S3: Mesenchymal self-condensation dynamics and morphology were influenced by the different sets of inhibitors (Related to Figure 3). (a) Self-condensation dynamics in the presence of inhibitors for cellular adhesion (EDTA), migration (paclitaxel), and contraction (blebbistatin). Here, the right panels show corresponding time-lapse montage across the center of the gels. Impact of blebbistatin onto (b) projection and (c) cross-sectional images of condensates on the soft and stiff region of the gels. Confocal fluorescence microscopy indicated that the blebbistatin significantly suppressed the concave morphology of condensates along the optical axis. Here yellow dotted line was displayed along the periphery of condensates in the cross-sectional images. (d) Corresponding schematic illustration of the morphology of condensates along the optical axis.

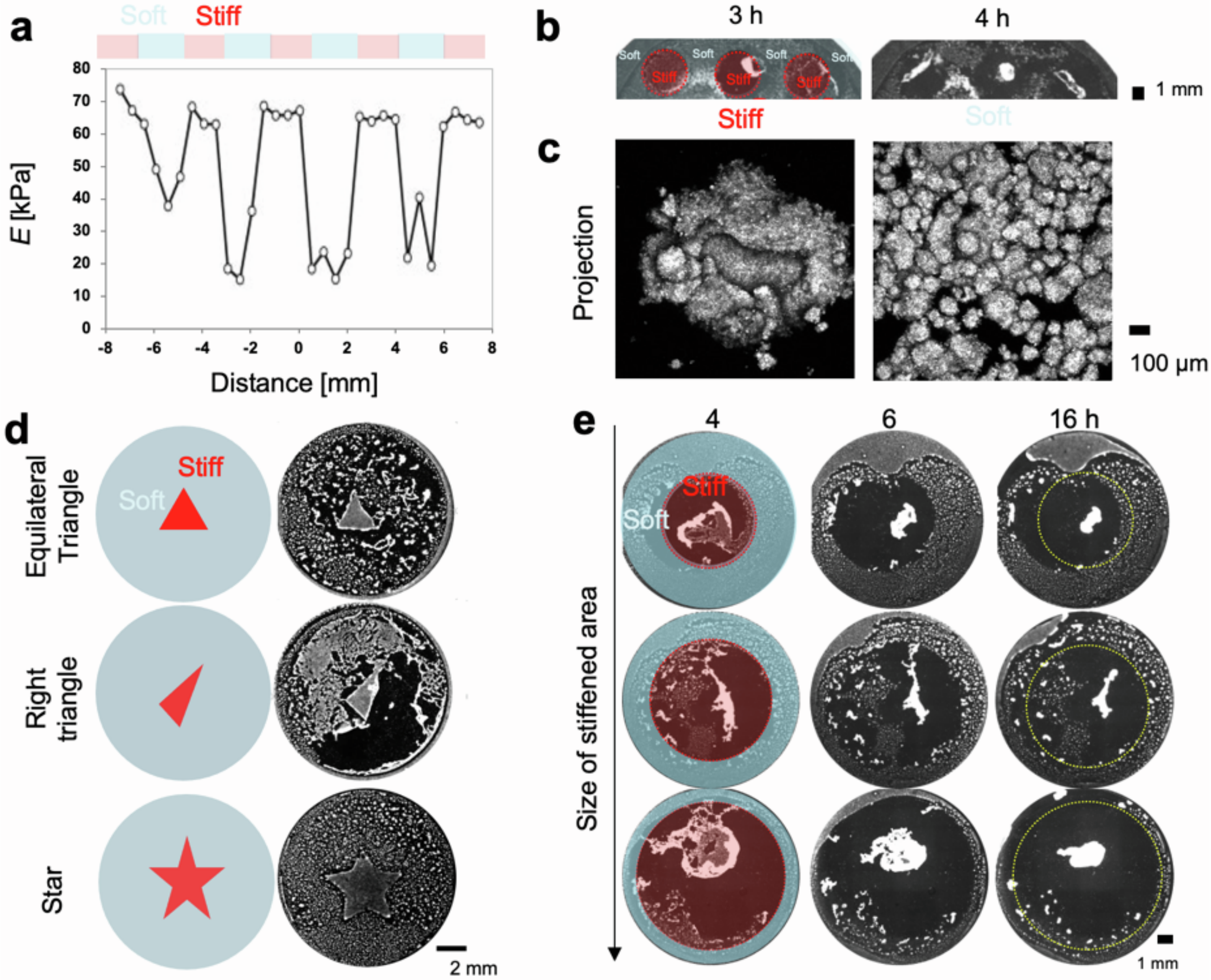

Figure S4. Impact of various mechanical patterns on controlling the morphology of condensates (Related to Figure 4). (a) Multi gradient mechanical pattern (soft-stiff-soft-stiff-soft) and (b) self-condensation of myoblast on the substrate. Representative confocal images of condensates on the stiff/soft region. Impact of shapes of mechanical patterns such as (d) equilateral/right triangle, star, and (e) circle with different diameters onto the self-condensation of myoblast.

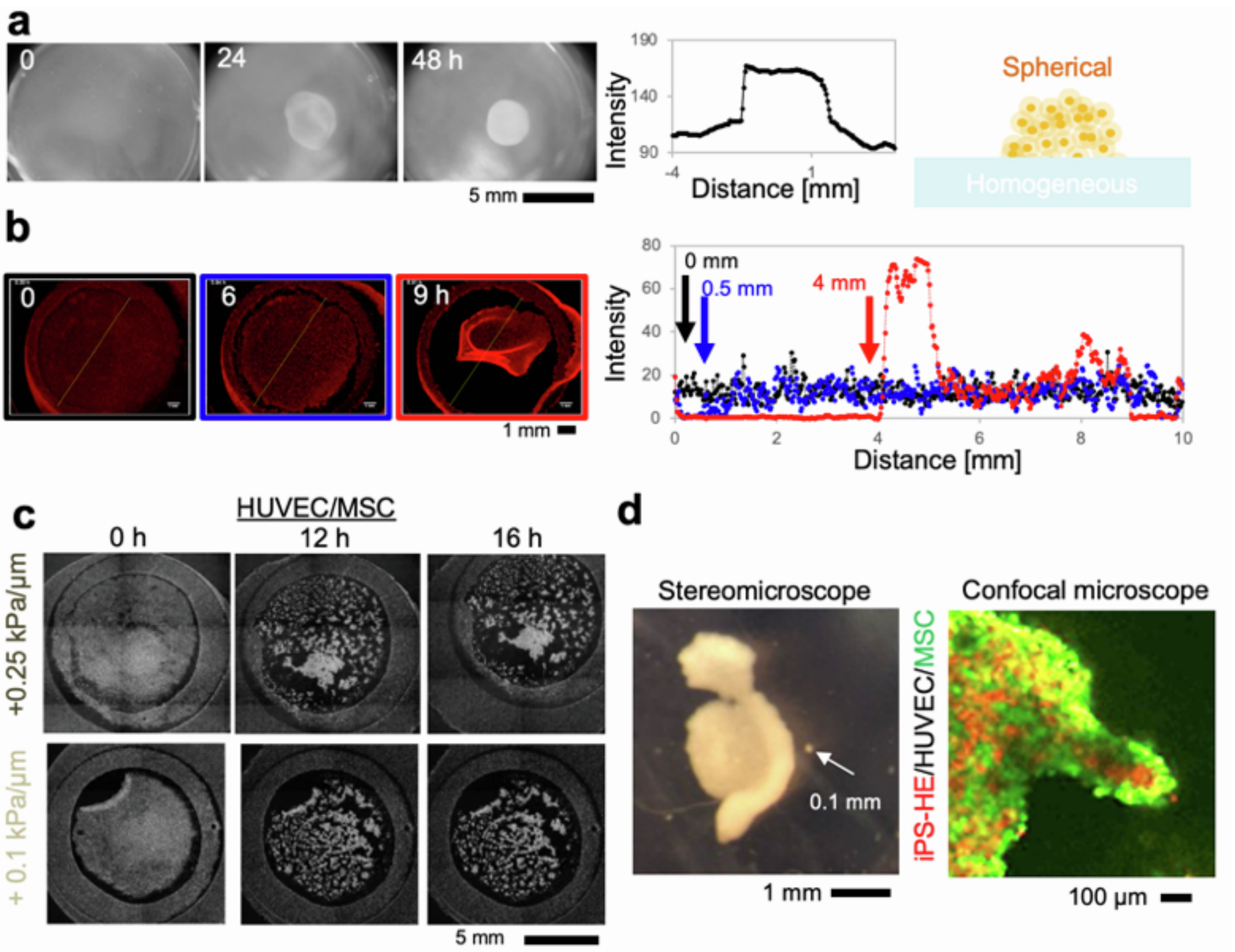

Figure S5: Local stiffening drives preparatory self-condensation of liver organ buds (Related to Figure 5). (a) Self-contraction of liver organ buds on homogenously soft gels (*i.e.,* Matrigel). (b) A Representative snapshot of MSC condensation on locally stiffened gels. (c) Impact of locally stiffened gels with high and low intrinsic mechanical gradient onto self-condensation of HUVEC/MSC condensates. A low mechanical gradient weakens the separation of cells at mechanical boundaries, and the single condensates were not formed on the stiff region. (d) Self-condensation of liver organ bud on locally stiffened gels (left panels show stereomicroscope images obtained by color CCD camera) and the confocal fluorescence images (right panels).
